# World Trade Center-Cardiorespiratory and Vascular Dysfunction: Assessing the Phenotype and Metabolome of a Murine Particulate Matter Exposure Model

**DOI:** 10.1038/s41598-020-58717-w

**Published:** 2020-02-21

**Authors:** Arul Veerappan, Assad Oskuei, George Crowley, Mena Mikhail, Dean Ostrofsky, Zakia Gironda, Sandhya Vaidyanathan, Youssef Zaim Wadghiri, Mengling Liu, Sophia Kwon, Anna Nolan

**Affiliations:** 1 Department of Medicine, Division of Pulmonary, Critical Care and Sleep Medicine, NYU, School of Medicine, NY, New York, NY USA; 2 Bureau of Health Services, Fire Department of New York, Brooklyn, NY USA; 3 Center for Advanced Imaging Innovation and Research (CAI2R) & Bernard and Irene Schwartz Center for Biomedical Imaging, Department of Radiology, NYU School of Medicine, NY, New York, NY USA; 4 Department of Environmental Medicine, New York University, School of Medicine, NY, New York, NY USA; 5 Department of Population Health, Division of Biostatistics, NYU School of Medicine, NY, New York, NY USA

**Keywords:** Diagnostic markers, Vascular diseases

## Abstract

Vascular changes occur early in the development of obstructive airways disease. However, the vascular remodeling and dysfunction due to World Trade Center-Particulate Matter (WTC-PM) exposure are not well described and are therefore the focus of this investigation. C57Bl/6 female mice oropharyngeally aspirated 200 µg of WTC-PM_53_ or phosphate-buffered saline (PBS) (controls). 24-hours (24-hrs) and 1-Month (1-M) after exposure, echocardiography, micro-positron emission tomography(µ-PET), collagen quantification, lung metabolomics, assessment of antioxidant potential and soluble-receptor for advanced glycation end products (sRAGE) in bronchoalveolar lavage(BAL) and plasma were performed. **24-hrs** post-exposure, there was a significant reduction in **(1)** Pulmonary artery(PA) flow-velocity and pulmonary ejection time(PET) (**2)** Pulmonary acceleration time(PAT) and PAT/PET, while (**3)** Aortic ejection time(AET) and velocity time integral(VTI) were increased, and (**4)** Aortic acceleration time (AAT)/AET, cardiac output and stroke volume were decreased compared to controls. **1-M** post-exposure, there was also significant reduction of right ventricular diameter as right ventricle free wall thickness was increased and an increase in tricuspid E, A peaks and an elevated E/A. The pulmonary and cardiac standard uptake value and volume 1-M post-exposure was significantly elevated after PM-exposure. Similarly, α-smooth muscle actin(α-SMA) expression, aortic collagen deposition was elevated 1-M after PM exposure. In assessment of the metabolome, prominent subpathways included advanced glycation end products (AGEs), phosphatidylcholines, sphingolipids, saturated/unsaturated fatty acids, eicosanoids, and phospholipids. BAL superoxide dismutase(SOD), plasma total-antioxidant capacity activity, and sRAGE (BAL and plasma) were elevated after 24-hrs. PM exposure and associated vascular disease are a global health burden. Our study shows persistent WTC-Cardiorespiratory and Vascular Dysfunction (WTC-CaRVD), inflammatory changes and attenuation of antioxidant potential after PM exposure. Early detection of vascular disease is crucial to preventing cardiovascular deaths and future work will focus on further identification of bioactive therapeutic targets.

## Introduction

The negative health effects of particulate matter (PM) exposure are a significant global burden. Epidemiologic studies demonstrate associations between PM exposure and development of lung and cardiovascular disease(CVD)^1,2^. Pulmonary vascular remodeling occurs in mild non-hypoxemic chronic obstructive pulmonary disease(COPD) patients^3^. People living near roadways and exposed to high PM_2.5_ have greater right ventricular(RV) mass and changes in RV function^4^. In a murine model of airway hyperreactivity, ambient PM exposure exacerbates pulmonary hypertension(PH)^5^. Furthermore, concentrated ambient air particles induce pulmonary vasoconstriction within 24-hours(24-hrs) post exposure^6^.

Our group has focused on the adverse health effects of World Trade Center (WTC)-PM exposure. WTC destruction caused an intense aerodigestive exposure of WTC-PM, leading to airway hyperreactivity and WTC-Lung Injury (WTC-LI; defined as forced expiratory volume in 1 second (FEV_1_) less than the lower limit of normal (LLN))^7–9^. However, association with CVD was noted in a few observational studies^10,11^. In addition, there were also increases in CVD-related hospitalizations after 9/11^12,13^. Pulmonary arteriopathy was noted in 58% of lung biopsies from WTC-exposed individuals^14^.

Evidence of WTC-Cardiorespiratory and Vascular Dysfunction(WTC-CaRVD) includes CVD risk factors, such as metabolic syndrome and pulmonary artery(PA)/aorta(A) ratio association with WTC-lung disease^8,9^. Cardiovascular biomarkers such as Apolipoprotein (Apo)-AII, soluble receptor for advanced glycation end-products(sRAGE) and soluble Vascular Cell Adhesion Molecule expressed shortly after WTC-PM exposure predict WTC-LI^11,15–17^.

Early diagnosis and therapeutic options are few, partly due to our limited understanding of PM-induced pathogenesis. While pulmonary vascular changes have been classically thought to occur due to the hypoxemia of late obstructive airway disease, the vascular hypothesis postulates that pulmonary vasculature remodeling leads to loss of lung function. These early vascular changes are critical to understand as they may be a therapeutic target to prevent development of airway and vascular diseases. Therefore, we hypothesized that a single exposure to WTC-PM would yield remodeling of the cardiovasculature.

**Figure 1:**
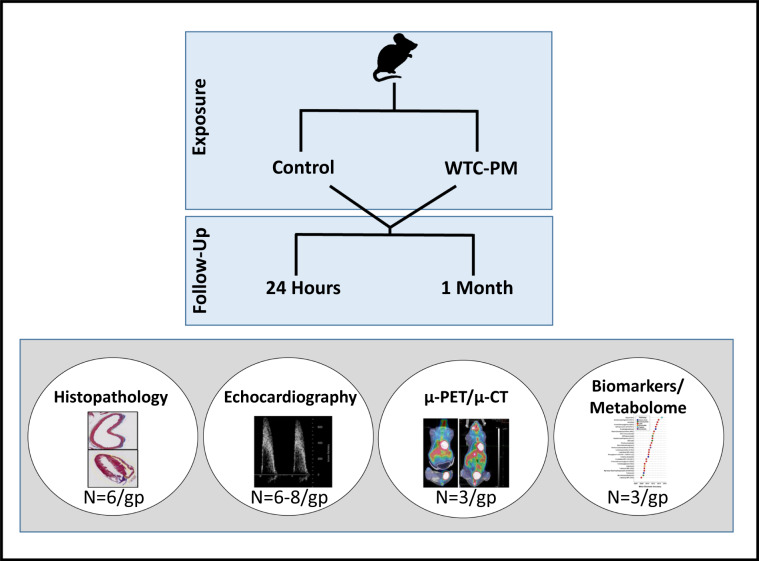
Study Overview. Mice were exposed to WTC-PM or PBS control, and had histologic, echocardiographic, radiologic and a metabolomic assessment at 24-hrs and after 1-M to assess PM exposure effects.

Endogenous repair and remodeling after WTC-PM exposure are critical endpoints that can be evaluated in murine models. We have demonstrated a murine model of WTC-LI, characterized by airway hyperreactivity and FEV_1_ loss, 24-hrs after WTC-PM exposure^16,18^. We will utilize this murine model to identify a cardiopulmonary disease-phenotype, WTC-Cardiorespiratory and Vascular Dysfunction (WTC-CaRVD), using histology, echocardiographic(Echo) and μ-PET/micro-computed tomography(μ-CT). We will also integrate imaging with histology, metabolomics, and quantification of antioxidant potential, Fig. 1.

## Results

### WTC-CaRVD has a distinct echo and µ-PET/µ-CT signature: complementary noninvasive multimodal imaging

**Figure 2:**
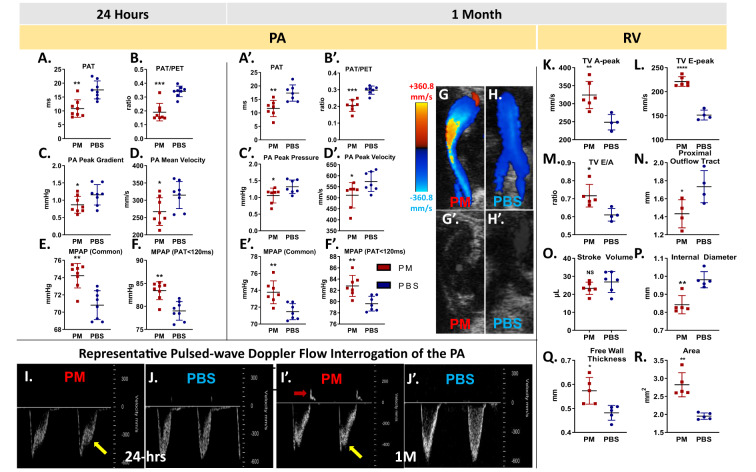
PA Echo Parameters. (**A**) PAT (p = 0.002, n = 8/group) (**B**). PAT/PET (p = 0.001, n = 8) (**C**). PA Peak Gradient (p = 0.050, n = 8) (**D**). PA Mean Velocity (p = 0.038, n = 8) (**E**). MPAP (Common) (p = 0.004, n = 7) (**F**). MPAP (PAT < 120 ms) (p = 0.002, n = 8) were all assessed after 24-hrs. (**A’**) PAT (p = 0.004, n = 7) (**B’**). PAT/PET (p = 0.001, n = 7) (**C’**). PA Peak Pressure (p = 0.034, n = 7) (**D’**). PA Peak Velocity (p = 0.038, n = 7) (**E’**). MPAP (Common) (p = 0.004, n = 7) (**F’**). MPAP (PAT < 120 ms) (p = 0.004, n = 7); were assessed after 1-M. (**G,H)** Bifurcated PA color flow Doppler (representative images) PM and PBS exposed respectively. Color Doppler mode shows flow direction of red (toward the transducer) or blue (away from transducer), with intensity as a function of velocity. (**G’,H’**) Structure of PA demonstrated by corresponding greyscale images. (**I,J** and **I’,J’**) Pulsed-wave Doppler waveforms (representative images) derived from these velocities are shown PM and PBS exposed both at 24-hrs and 1-M, respectively. A notch (yellow arrow) appears during pulmonary ejection time at both 24-hrs and 1-M after PM-exposure that is not seen in PBS. Further, a bidirectional flow pattern appeared at 1-M, indicating a pulmonary valve insufficiency (red arrow). RV Echo Parameters. (**K**) A-peak of tricuspid valve (p = 0.010, n = 6). (**L**) E-peak of tricuspid valve (p = 0.010, n = 6). (**M)** E/A ratio of tricuspid valve (p = 0.016, n = 6). (**N**) Proximal outflow tract (p = 0.045, n = 4), (**O**). Stroke Volume (p = 0.237, n = 6) (**P**). Internal diameter (p = 0.002, n = 5) (**Q**). Free wall thickness (p = 0.017, n = 5), (**R**). RV Area (p = 0.003, n = 5). *p < 0.05, **p < 0.01, ***p < 0.001, ****p < 0.0001.

Echo was utilized to measure pulmonary velocity through pulmonary Doppler flow as an indirect measurement of pulmonary vascular resistance and assessing for PH in PM and PBS controls, Fig. 2. Echo parameters were derived from Doppler flow images and shown in Fig. 2A–F. Representative images shown in Fig. 2G,H denotes color mode whereas Fig. 2G’,H’ denotes corresponding greyscale images. Pulmonary artery waveforms at 24-hrs and 1-M showed a change in flow, Fig. 2I,J,I’,J’. We noted a significant reduction in PA flow (velocity) and presence of a midsystolic notch during the PET after PM-exposure (Fig. 2I, yellow arrow) when compared to PBS controls, Fig. 2J, and persisted at 1-M, Fig. 2-I’,J’. In addition to the midsystolic notch, a bidirectional flow pattern appeared that may indicate pulmonary valve insufficiency occurred 1-M after PM exposure, Fig. 2I’, red arrow) when compared to the PBS control, Fig. 2J’.

In models of PH, PAT is negatively correlated with pulmonary vascular resistance. Doppler showed that the PAT, PAT/PET, peak gradient, and mean velocity were significantly reduced in WTC-PM compared to controls at both 24-hrs and 1-M, Fig. 2A–D,A’–D’. MPAP was similarly increased in PM-exposed mice compared to PBS again at both time points, Fig. 2E,F,E’,F’. This could reflect PA dysfunction in the setting of acute injury at 24-hrs and wall stiffness and/or remodeling after 1-M. However, the PA internal diameter was not significantly different between PM and PBS exposed mice at 1-M (Mean (mm) ± SD, 1.13 ± 0.05 PM, 1.10 ± 0.04 PBS).

In line with these findings, the tricuspid valve (TV) E and A wave peaks as well as E/A peak ratio, both indicators of RV diastolic filling, were significantly elevated in PM-exposed mice after 1-M, Fig. 2K–M. These results indicate an alteration in TV function at 1-M after WTC-PM exposure. The proximal RV outflow tract was decreased, Fig. 2N, whereas the distal (Mean (mm) ± SD, 1.16 ± 0.12, 1.31 ± 0.21) and total RV outflow tract (1.96 ± 0.11, 1.84 ± 0.15) were not significantly different between PM and PBS exposed respectively. RV stroke volume is maintained, Fig. 2O. Additionally, the RV internal diameter was significantly reduced, whereas free wall thickness and area were increased, indicating possible RV hypertrophy after PM exposure, Fig. 2P–R.

**Figure 3:**
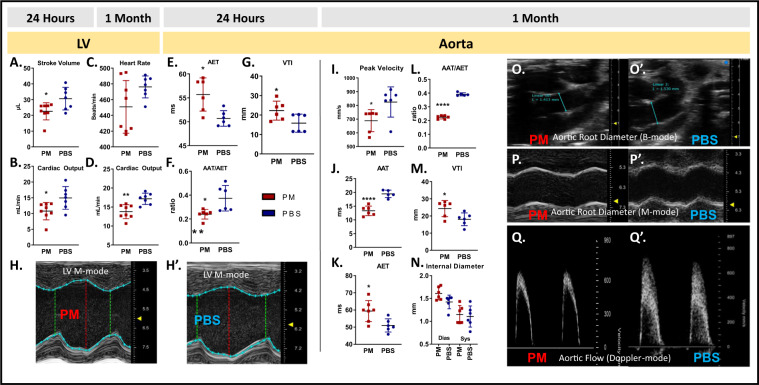
LV Echo measures after 24-hrs: (**A**) Stroke volume (p = 0.034, n = 8) (**B**). Cardiac output (p = 0.030, n = 8), and at 1-M: (**C)**. Heart rate (p = 0. 0.244, n = 8) (**D)**. Cardiac output (p = 0.004, n = 8). Aortic Echo Measures after 24-hrs: (**E**). AET (P = 0.017, n = 6), (**F**). AAT/AET ratio (p = 0.007, n = 6), (**G**). Aortic VTI (P = 0.041, n = 6). **(H,H’**) LV in M-mode (representative images) for PM and PBS exposed after 1-M, respectively. Aortic measures after 1-M: (**I**) Aortic peak velocity (p = 0.022, n = 6), (**J**). AAT (p = 0.004, n = 6), (**K**). AET (p = 0.0163, n = 6), (**L**) AAT/AET (p = 0.004, n = 6), (**M**) VTI (p = 0.030, n = 6) and (**N)** Internal diameter (P = 0.565, n = 5). Aortic Imaging (representative) **(O)**. Root diameter (B-mode) (**P**). Root Diameter (M-mode) **(Q)**. Aortic flow (Doppler mode) 1-M after PM exposure in comparison to PBS; (**O’**,**P’**,**Q’)**, respectively. *p < 0.05, **p < 0.01, ****p < 0.0001.

In evaluation of the left ventricle (LV), measures of cardiac function, stroke volume and cardiac output were significantly decreased in PM mice after 24-hrs compared to controls, Fig. 3A,B. These measures did not vary with heart rate at either time point, Fig. 3A–D. Aortic Doppler measurements of AET, VTI, and AAT/AET changed after 24-hrs, and persisted at 1-M, indicating possible aortic stiffness and blood flow reduction, Fig. 3E–G,I–M. Aortic internal diameter in systole and diastole were no different between the two groups, Fig. 3N.

Representative images of the mouse LV in M-mode are shown 1-M after PM and PBS-exposures, Fig. 3H,H’, the aortic root in B-mode and M-mode, Fig. 3O,P,O’,P’, and aortic flow (Doppler mode), Fig. 3Q,Q’ respectively. A complete listing of echocardiographic measures may be found in Supplemental Table 1.

### µ-PET/µ-CT

**Figure 4:**
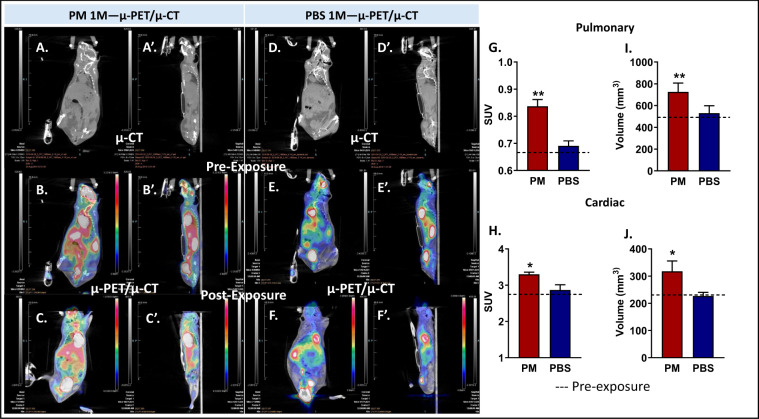
Cardiopulmonary µ-PET/µ-CT Assessment. Coronal Pre and Post PM and PBS exposure (**A,D**). µ-CT sections; (**B,E**). µ-PET/µ-CT Pre Exposure and (**C,F**). Post Exposure µ-PET/µ-CT. (**A’–F’**) Sagittal µ-PET/µ-CT sections of each of the above exposures and imaging modalities respectively. SUV of Assessment 1-M post exposure G. Lung (**p < 0.01) (**H**). Cardiac (*p < 0.05) (**I**). Lung volume (**p < 0.01) (**J**). Cardiac Volume (*p < 0.05), n = 3/group. Mean baseline SUV of the lung and heart indicated with a dashed line. Error bars represent mean ± SD.

Representative coronal and sagittal µ-PET/µ-CT images of baseline and 1-M after exposure to WTC-PM or PBS are shown, Fig. 4A–F,A’–F’. After 1-M, the pulmonary and cardiac standard uptake value (SUV) in PM-exposed mice were significantly elevated compared to controls, Fig. 4G,H. Volume of pulmonary and cardiac tissue in PM-exposed mice was significantly elevated compared to controls, Fig. 4I,J. SUV and volume were not different between pre- and post- exposure of PBS.

### WTC-CaRVD has a distinct collagen deposition pattern and α-SMA expression

**Figure 5:**
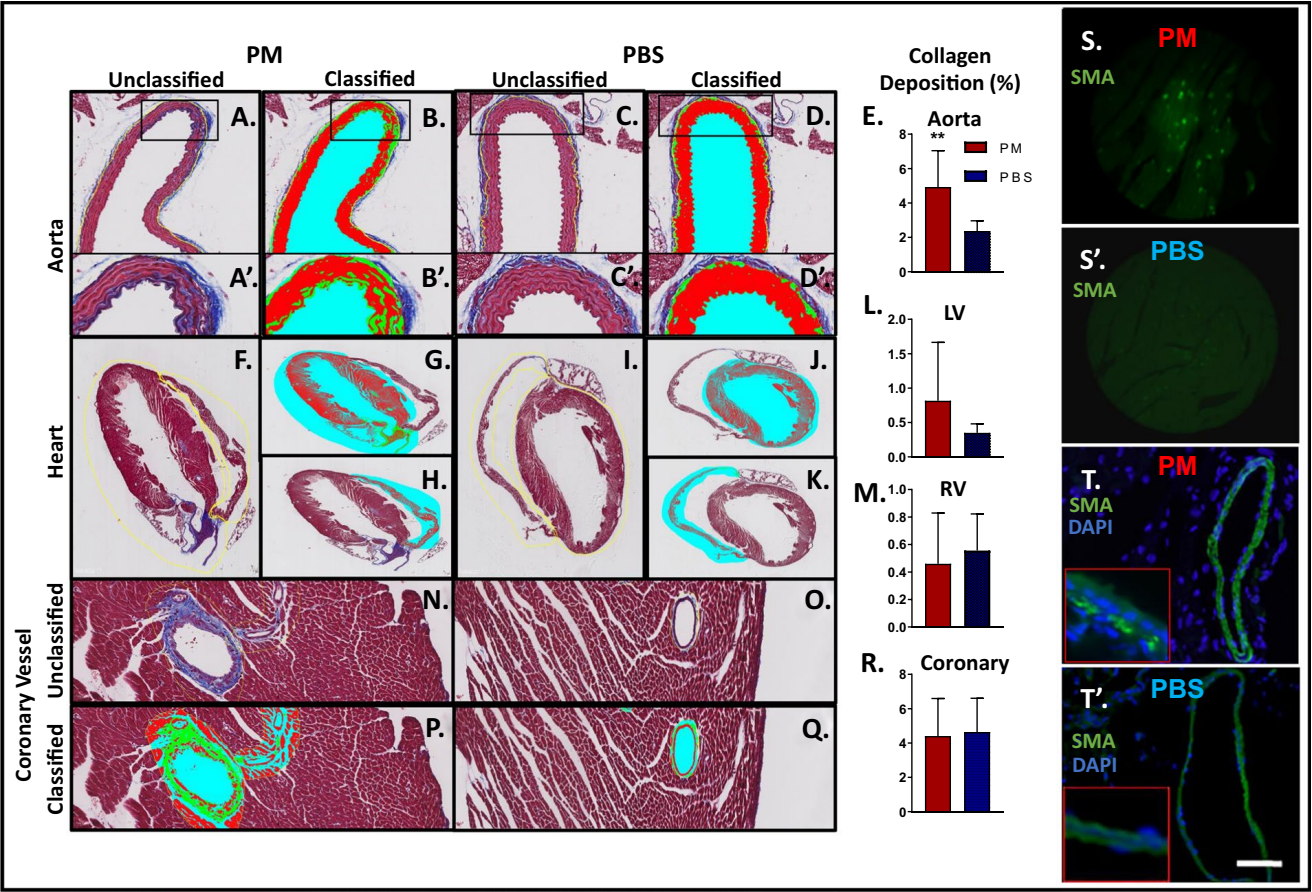
Cardiovascular Histology. Collagen Assessment of (**A–D)** (10×). Thoracic Aorta after PM and PBS exposure respectively (representative images of unclassified and classified sections shown) (**A’–D’)**. Representative images at 40×. (**E**) Percent Collagen Deposition of the aortic sections, *p < 0.05 (**F,I)**. Representative Unclassified cardiac sections after PM and PBS exposure respectively **(G,J)**. Classified images of LV after PM and PBS exposure respectively **(H,K)**. Classified images of RV after PM and PBS exposure respectively. (**L,M**) Percent collagen deposition of LV and RV (**N–Q**). Unclassified and classified sections of coronary vessels of PM-exposed and PBS at 40×. (**R**) Percent collagen deposition of coronary vessel (**S,S’**). Representative images of Murine cardiac tissue (20×) α-SMA (green) expression with PM and PBS exposure, (**T,T’**). Lung vasculature (PA) α-SMA expression at 20× and inset at 40× of PM and PBS exposure. Scale bar equals 100 µm and co-stained with DAPI (blue) in lung for nuclei visualization.

Collagen deposition is one of the body’s responses to injury and inflammation. Global collagen deposition noted on histopathology was assessed in our murine model of PM-exposure, Fig. 5. The aorta had significantly greater collagen in PM-exposed mice relative to PBS after 1-M, Fig. 5A–E. We quantified collagen deposition on left and right ventricles of the heart, Fig. 5F–M. There was no significant difference in collagen deposition between the LV and RV walls of PM- and PBS-exposed mice. We also quantified coronary vessels within cardiac walls, but saw no significant difference in collagen deposition between PM-exposed mice and PBS controls, Fig. 5N–R. *Classifier Performance* Our cardiac and aortic models demonstrated median accuracies of 92.5% (85.0–95.0) and 90% (85.0–90.0), respectively, as assessed by blinded investigators.

α-SMA is widely used as a marker of activated fibroblasts. Our assessment of the heart in WTC-PM exposed mice at 1-M revealed elevated α-SMA expression compared to controls, Fig. 5S,T,S’,T’. Our results suggest a positive correlation between α-SMA and collagen expression in PM exposed tissue, indicating that α-SMA may have a possible role in cardiopulmonary tissue fibrosis.

### WTC-CaRVD is associated with an augmentation of metabolites and oxidative stress mediators

#### Metabolomics

**Figure 6:**
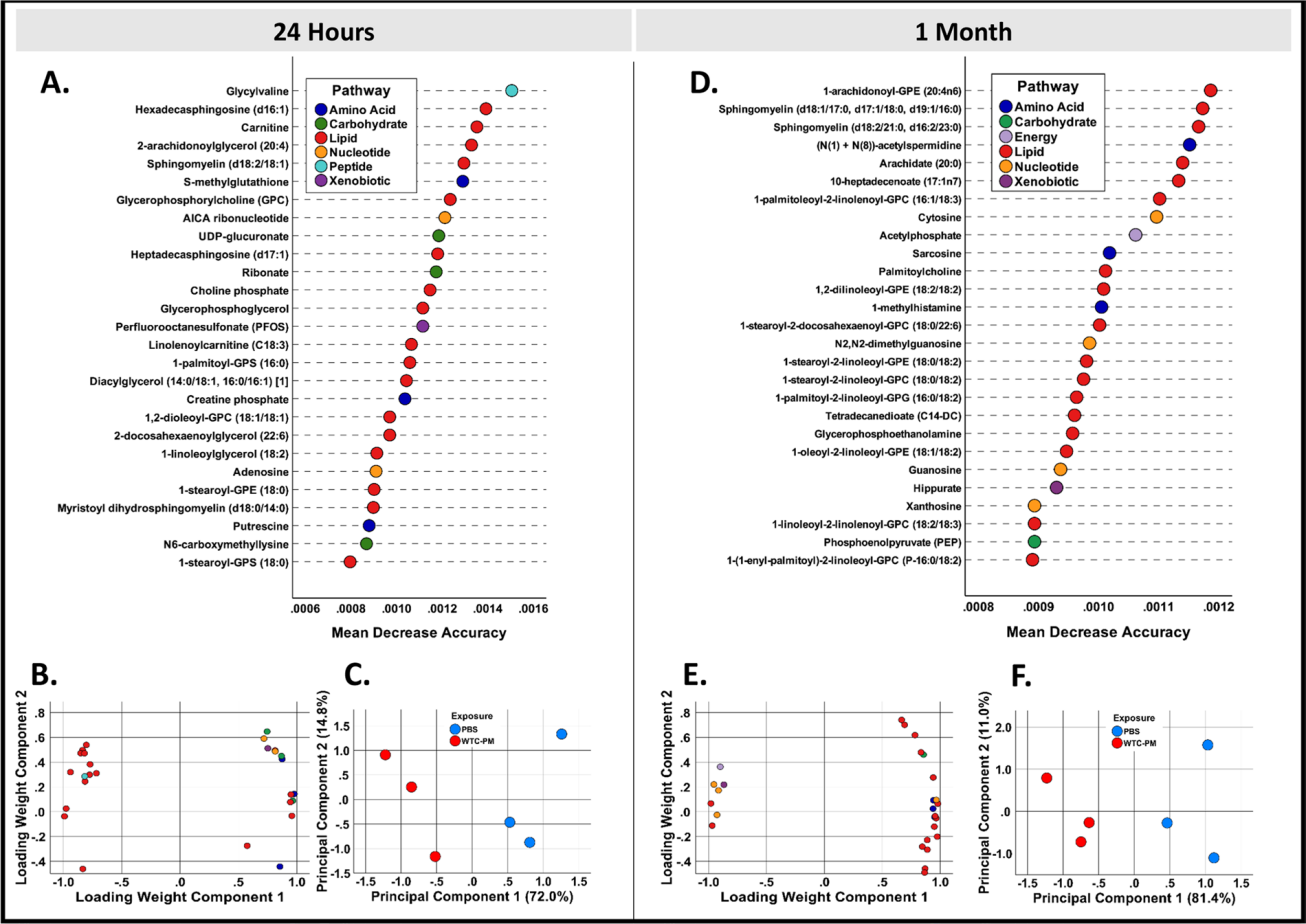
Metabolomic Profiling at 24-hrs and 1-M (**A,D**). RF Analysis of Refined Metabolic Profile performed after 24-hrs and 1-M respectively. Variable importance in projection is measured by mean decrease accuracy; the top 5% of metabolites important to class separation are shown (**B,E**). PCA loading weights plot of refined metabolic profile (**C,F**). PCA scores plot of refined metabolic profile reveals class separation between PM- and PBS-exposed mice.

We assessed the metabolome of mice after PM-exposure. Of 733 metabolites detected, 542 qualified and were included in further analysis. We identified refined profiles of metabolites with the highest mean decrease accuracy after 24-hrs and 1-M, Fig. 6A,D. Random Forest (RF) of the refined metabolite profiles achieved 0% out-of-bag estimated error rates (100% estimated accuracy). PM-exposure exhibited a persistent metabolome in mice 24-hrs and 1-M. Prominent pathways included AGEs and known mediators of lung disease such as phosphatidylcholines, sphingolipids, saturated and unsaturated fatty acids, eicosanoids, and phospholipids at both 24-hrs and 1-M. These metabolites were previously identified in firefighters that developed WTC-LI^17^. Principal component analysis (PCA) of the refined metabolite profiles of 24-hrs and 1-M captured 93.2% and 97.1% of variance, respectivley, in the 3 components retained based on examination of the scree plots. In the PCA loading weights plots, we see clusters of highly correlated lipid and amino acid subtypes, Fig. 6B,E. We also demonstrate class separation in components 1 and 2, Fig. 6C,F.

**Figure 7:**
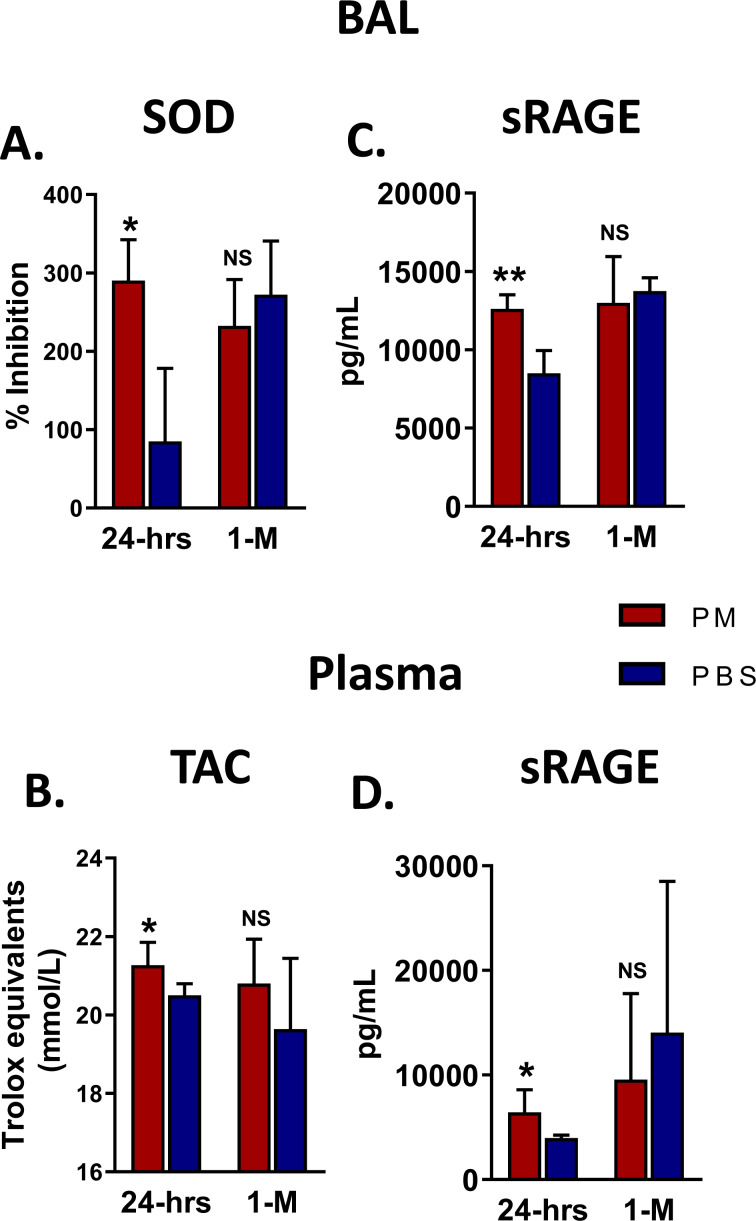
Antioxidant potential of murine BAL/plasma, 24-hrs and 1-M after PM exposure. (**A**) SOD Activity in BAL significant increased as compared to PBS controls 24-hrs after PM exposure (*p = 0.016) but not after 1-M. (**B**) TAC in plasma after PM exposure was significant increased 24-hrs exposure (*p = 0.030) but not after 1-M. (**C**) sRAGE in BAL was significantly increased 24-hrs after exposure but not after 1-M; **p = 0.008. **(D)** sRAGE in plasma was significantly increased only 24-hrs after PM exposure. *p = 0.030. n = 5/group. *p < 0.05, **p < 0.01 and NS = not significant.

**SOD** and Total antioxidant capacity assay (**TAC)** were significantly increased in BAL fluid and plasma 24-hrs after PM exposure compared to PBS but not after 1-M, Fig. 7A,B. Similarly, **sRAGE** was significantly increased in both compartments, Fig. 7C,D. However, there were no differences in these biomarkers 1-M post-exposure. These results suggest that oxidative stress mediators may participate in the pathogenesis of WTC-PM induced cardiopulmonary dysfunction and injury.

## Discussion

The “Vascular Theory” hypothesizes that changes in the vascular bed may contribute to the development of airways disease. Pulmonary perfusion abnormalities, reduced pulmonary microvascular blood flow, and reduced blood return to the heart occur prior to development of abnormal lung meachanics^19–21^. Our study shows that a single exposure of WTC-PM is associated with acute and chronic cardiopulmonary changes. WTC-PM exposure has traditionally been known to cause pulmonary disease^7–9^. We demonstrate that there are early vascular and hemodynamic changes, evidence of hypermetabolic activity, collagen deposition, and oxidative stress following WTC-PM exposure. Furthermore, a single exposure of WTC-PM leads to persistent elevation of intracardiac pressures. Strikingly, a rise in MPAP and PAT/PET ratio, as well as a drop in mean PA velocity, occur as early as 24-hrs after WTC-PM exposure. There is evidence that PM exposure results in vascular endothelial damage and impaired vasodilation just hours after exposure^22^.

Increased RV free wall thickness could be one of the reasons for reduced internal diameter. These changes in the PM-exposed mice are suggestive of RV hypertrophy and diastolic dysfunction. This was also supported by findings of elevated TV E and A wave peaks. These changes suggest increased pressures in the PA and possible incipient pulmonary hypertension within 24-hrs post-exposure and persists at 1-M. Another well-established indicator of elevated pulmonary vascular resistance is the presence of notching or systolic deceleration in the Doppler PA flow wave. Mid-systolic notching correlates with the highest pulmonary vascular resistance compared to late-systolic or absent notching^23^. PM-exposed mice had mid-systolic notching at 24-hrs and late-systolic notching at 1-M, suggesting pulmonary vascular resistance peaks early after PM exposure and subsequently decreases but remains abnormal. In addition to the late-systolic notching, a bidirectional flow pattern appeared at 1-M. This could indicate pulmonary valve insufficiency, suggesting pulmonary vascular disease progression. Normally, the Doppler Echo pattern of PA systolic flow is symmetric, while in pulmonary hypertension, the Doppler flow becomes asymmetric, with the peak velocity occurring earlier and PAT is decreased by premature pulmonary valve closure due to high PA pressure. In addition, the reduced PAT also leads to a decreased PAT/ET. The normalization of PAT by PET offsets several confounders such as heart rate^24^ and cardiac output^25^ that might independently affect PAT. Although WTC-PM exposure did not affect heart rate, cardiac output and stroke volume were decreased. Doppler Echo of PA showed that exposure to WTC-PM decreased PAT by 33% and the ratio of PAT/ET by 38%, resulting in asymmetric flow compared to PBS, and suggesting that WTC-PM exposure may contribute to premature pulmonary valve closure. A number of studies have demonstrated pulmonary vascular remodeling, such as smooth muscle hyperplasia and thickened intima of pulmonary arterioles, in COPD patients, as well as smokers without obstruction^3^, suggesting that patients with significant PM exposure who have yet to develop airflow obstruction may be undergoing similar vascular remodeling.

Given the prevalence of hypertension and increased rate of heart failure exacerbation in WTC first responders, we sought to assess the LV and systemic vasculature in our murine model. Interestingly, in PM-exposed mice, the LV stroke volume and cardiac output were significantly reduced 24-hrs post-exposure. In addition, our results showed decreased AAT and AAT/AET, and increased AET and AVTI, suggesting impaired aortic function in mice 24-hrs and 1-M post-PM exposure. Prior murine studies have shown that PM exposure leads to reduced myocardial contractility, with potential mechanisms involving: (1) Ca^2+^ handling in cardiac myocytes and proteins involved in Ca^2+^ sequestration; (2) collagen deposition, ventricular wall thickening, and loss of elasticity; (3) altered cellular pathways resulting in nitric oxide synthase uncoupling and subsequent reactive oxygen species production^26^.

Parallel to these physiologic changes, we sought to identify concurrent inflammation within the lungs and heart. Acute and chronic inflammation can be caused by PM by release of pro-inflammatory chemicals into adjacent vessels and lymphatic tissue^5^. Increased neutrophilic rolling and adhesion to the microvascular wall follows^22^. µ-PET imaging has traditionally been used to detect hypermetabolic areas within the organs, primarily to assess malignancy. However, as prior observational studies show, µ-PET/µ-CT imaging can detect inflammatory conditions such as sarcoidosis or coal workers’ pneumoconiosis^27^. We were able to measure whole lung and heart SUV 1-M after WTC-PM exposure, and results suggest persistent inflammation and/or hypermetabolic cellular activity. Prior studies have shown that pre-pulmonary hypertension rats exhibit higher ^18^F-fluorodeoxyglucose (FDG) avidity, which decreases with treatment. As pulmonary hypertension progresses, there appears to be a metabolic shift toward inflammation-associated glycolysis, oxidative stress, and fibrosis^28^. Our echo and µ-PET/µ-CT data suggests that an increased volume of heart 1-M after PM exposure could be from possible RV enlargement and hypertrophy. Similarly, the lung volume was increased in PM-exposed mice. This could be due to hyperinflation from obstructive pulmonary diseases such as COPD and asthma^29,30^.

The underlying pathways associate with WTC-CaRVD have not been previously studied. The generation of reactive oxygen species and cellular responses to oxidative stress after WTC-PM exposure may contribute to tissue injury and WTC-CaRVD pathogenesis. The BAL SOD level was higher in our WTC-PM mice compared to controls 24-hrs post exposure. Animal PH models demonstrate lower levels of SOD and higher superoxide anions within the smooth muscle and adventitia of PA^31^. Intratracheal administration of recombinant human SOD selectively decreases PA pressures and increases vascular response to NO^32^.

Oxidative stress initiates pro-apoptotic signal transduction cascades and inflammatory mediator release, leading to cell death, especially in endothelial cells. Endothelial cell damage and death is a key event in the development and worsening of vascular pathologies. In our experiments, PM-exposed mice demonstrate this injury by upregulation of α-SMA expression and focal collagen formation in vascular and cardiac tissue, especially within the coronary arteries. Findings of increased BAL SOD and plasma TAC in PM-exposed mice indicate an area of future research into oxidative stress mechanism.

Our recent study of the WTC-exposed firefighter metabolome suggested that amino acid and lipid metabolites are the most active in WTC-LI pathogenesis^17,33^. To investigate whether these metabolites are adversely altered in an animal model that mimics human WTC-PM exposure, we assessed the global metabolome of PM-exposed mice and PBS controls. Our refined profile of most discriminative metabolites included a majority of lipid metabolites, followed by several amino acid, carbohydrate, and nucleotide metabolites at 24-hrs post exposure. Further, we identified important lipid, nucleotide, and amino acid metabolites in mice 1-M post exposure. We noted an upregulation of several lipid metabolites in PM-exposed mice.

The effect of the lipidome on systemic vasculature is crucial, and the change in the PM-exposed mouse lipid profile may be contributing to their cardiopulmonary vascular dysfunction. Literature suggests a causative role of oxidized lipid metabolites in the initiation and progression of vascular remodeling via oxidative stress^34^. Nevertheless, significant questions remain, including which mechanisms are most important for driving oxidative stress, what treatment strategies can effectively reduce oxidative stress, and what are the key components of oxidized lipid metabolites that drive WTC-PM vascular remodeling and injury.

Our present study also demonstrates that PM-exposed mice had significantly elevated sRAGE in BAL and plasma which is in line with our prior findings that RAGE is pivotal to WTC-PM lung injury^16^. RAGE has been implicated in cardiopulmonary diseases, such as pulmonary arterial hypertension^35^. Further, RAGE is overexpressed in smooth muscle cells of patients with both idiopathic and heritable pulmonary arterial hypertension. An independent study showed that EN-RAGE augments chronic kidney disease-triggered osteogenesis in murine vasculature, similar to features of enhanced vascular calcification in patients with chronic and end-stage kidney disease^36^. The RAGE/AGE signaling has been implicated in the development of fibrosis and collagen formation^37^.

### Limitations

This investigation has several limitations. We have focused on early vascular changes that may be related to lung disease. Future work will focus on vascular changes that may be related to clinical end-organ pathology such as cardiac and cerebral ischemia. Also, serial dose response was not investigated, and only a single dose of PM aspiration was used. The dosage was based on earlier work aiming to replicate the acute oropharyngeal aspiration of WTC-PM by rescue workers during the events of 9/11. A murine oropharyngeal aspiration of 200 µg of WTC-PM is estimated to be equivalent to a human exposure to 850 µg/m^3^ over an 8-hour work period^18^. This dose represents exposure on the lower end of intensity likely experienced at the site, as the PM concentration is estimated to have reached the thousands of µg/m^3^ range immediately post-WTC collapse^38,39^.

Finally, although we have identified elevated sRAGE and anti-oxidant potentials followed by cardiovascular dysfunction and injury, additional pathways responsible for adverse cardiopulmonary effects of WTC-PM insult remain unidentified. Finally, we have used noninvasive imaging to measure cardiopulmonary vascular functional variabilities for this study. However, invasive methods using right heart catheterization may be needed to accurately measure RV pressure.

### Conclusions

In summary, our results demonstrate that the acute cardiovascular effect of WTC-PM exposure is persistent and may lead to cardiopulmonary dysfunction, inflammation, and remodeling. Our study suggests that in a murine model, WTC-PM exposure induces oxidative stress and RAGE expression and results in functional vascular dysfunction and tissue remodeling. Specifically, exposure was associated with changes of the PA, aortic, and ventricular hemodynamics. Furthermore, this work implicates WTC-PM as a culprit of WTC-CaRVD, and that targeting oxidative stress mediators including RAGE may be a biologically plausible therapeutic targets. This work can be extended to the global health concerns of those with PM exposure and concomitant cardiovascular disease.

## Methods

### Research animal subject ethical statement

All experiments were performed in accordance with NIH guidelines, and as approved by the New York University (NYU) School of Medicine’s Institutional Animal Care and Use Committee (IACUC), protocol # 16-00447 and the Animal Welfare Assurance # D16-00274. Experiments were conducted in accordance with ethical guidelines outlined by NYU and the NIH.

### Murine oropharyngeal PM aspiration model

C57Bl/6 mice (Jackson Laboratory) aged 8–10 weeks, aspirated 200 µg of WTC-PM_53_ (from 5 locations within 0.5 miles of Ground Zero 9/13/01) suspended in sterile PBS or PBS control, Fig. 1^16,18^. Oropharyngeal aspiration is considered to be superior to inhalational methods of exposure to particulate matter, and has been successfully used in prior work in murine models of PM-exposure^16,18^. Mice had free access to food/water and 12-hour light/dark cycles.

Imaging was performed by an investigator blinded to group assignment, 24-hrs and 1-M after exposure. Heart rate monitored, using EKG electrodes(Parker Labs), was maintained at ~500 beats/minute by titrating isoflurane while on a heated plate (37 °C) and monitored via rectal thermometer. Anesthesia delivered in a sealed chamber with 2%-isoflurane and sustained with 1.5%-isoflurane/100% supplemental oxygen via nose cone^40^.

i. ***Echocardiography*** **was** performed on a VisualSonics Vevo 3100 ultrasound scanner (Fujifilm) equipped with a 40-MHz probe (MX 550D; n = 8/group)^41^. Chest hair was removed (Nair) and ultrasound gel was applied (Aquasonic), B (brightness)- and M (motion)- mode and pulse wave Doppler images in parasternal long/short axis views were obtained. MPAP, PET, PAT, pulmonary VTI, PA diameter, RV free wall thickness, RV stroke volume, RV outflow tract, proximal RV outflow tract diameter, distal RV outflow tract diameter, AAT, AET, aortic VTI, LV dimensions were measured (end diastolic and systolic diameters, (LVEDd, LVEDs)). All Doppler data for PA and aorta were averaged from at least 3 consecutive beat cycles. Three loops of M-mode data for LV and RV were captured for each animal, and averaged from at least 5-beat cycles/loop. Parameters were detected using the American Society for Echo leading-edge technique allowing for the determination of LV percent fractional shortening (%fractional shortening = [(LVEDd − LVEDs)/LVIDd*100]).
ii. **µ-PET/µ-CT**. Mice (n = 3/group) were fasted for at least 8 hours. Each subject was injected with 200 µL of ^18^F-FDG in PBS via the tail vein using a PHD 2000 computerized syringe pump (Harvard Apparatus). The injected doses ranged from 3.7 to 7.4 MBq (100 µCi to 202 µCi) using a 150 µL/min infusion rate; mice were imaged on a Siemens Inveon MM scanner, equipped with a µ-PET detector ring comprising of 16 detector blocks each containing a row of four lutetium oxyorthosilicate detectors for a total of 64 detectors capable of 1.4-mm isotropic spatial resolution. The scan consisted of a 60 minute whole body µ-PET acquisition after the injection of ^18^F-FDG, followed by a 100-µm µ-CT scan after each µ-PET acquisition to assess the attenuation correction for the ^18^F-FDG datasets. M2M BIOVET module and software was used to confirm vitals throughout the scan. The 60 minute dynamic acquisition of the µ-PET data was reconstructed into 3D sinograms (Open Inveon Research Work) with the following frames: 12 frames of 10 seconds, 16 frames of 30 seconds, 10 frames of 60 seconds and 8 frames of 300 seconds. SUV and mean FDG uptake were normalized to body weight, injected dose, and radioactive decay assessed. The average SUVs of each subject over 60 minute time period was chosen for comparison. This period was chosen as FDG uptake is stable and consistent with the SUV profiles of the datasets. Organ volume was estimated by a blinded investigator drawing a region of interest around the heart and lung captured on µCT^42^.

### Histology

Dedicated mice (n = 6/group) had their hearts and aortas fixed in 4%-paraformaldehyde and embedded in 10%-paraffin. 5-*μ*m sections were stained with hematoxylin/eosin for morphometry and Gomori Trichrome(Thermoscientific) for collagen. Collagen deposition was assessed with Orbit Image Analysis (http://www.orbit.bio/)^43^. Classification models were trained on whole aorta and heart images(Nanozoomer-2.0HT;2 sections/slide) using manual annotations. 3,700 and 2,300 manual annotations of the heart and aorta were made respectively(2 sections/mice; n = 6 mice per group). Individual coronary vessels within the cardiac wall were assessed (2 sections/mice; n = 3 mice per group). Manual annotations were made to include collagen deposition within the aortic wall, excluding the tunica adventitia^44^. Proportion of collagen deposition to entire classified area within the region of interest was quantified(%). Model performance was assessed independently by three blinded and trained investigators. The same three randomly selected sub-images of each slide were rated on a scale of 1–10 (10—most accurate) for classification accuracy based on the Gomori Trichrome staining.

### Immunohistochemistry

Tissue were fixed *in situ* with 4% paraformaldehyde (Sigma), stored in 70% ethanol (4 °C) and were then processed through a series of graded ethanol, from 70% to 100%, Xylene, and paraffin (Leica Peloris). Heart and vessels (n = 6/group) were sectioned at 5 µm onto charged slides using a rotary microtome as previously described^18,45^. Sections were stained with hematoxylin/eosin for primary assessment of structural architecture and Gomori Trichrome for assessment of collagen deposition. The stained slides were then digitally scanned (Slidepath, Leica). Investigators were blinded to experimental condition during selection and measurement of all fields^16,18,45^. Immunohistochemistry (Nikon Eclipse fluorescence upright microscope and processed with NIS Elements Basic Research Microscope Imaging Software) of 5-µm sections of mouse heart and lung was performed using 1°-antibodies, anti-α-SMA (1:200), corresponding 2°-antibodies (1:250) and DAPI (1:10,000) (SantaCruz) performed (Olympus-BX51/NikonD5100).

### Assessment of oxidative stress

**i**. ***SOD activity*** was assessed in the plasma (Abcam) as per the manufacturer’s protocol (n = 5/group). **ii.** TAC was determined in plasma(Cayman chemical). **iii. sRAGE** was quantified in both plasma and BAL(R&D). **iv**. ***Metabolomics****.* 100 mg of lung was snap frozen at −80 °C (n = 3/group) and global metabolome assessed (Metabolon)^17^. Controls were analyzed with the samples: a technical replicate, blanks, and quality check standards were assayed. Compounds underwent quality control, curation, and were matched to library entries of retention index, mass, and spectral data.

### Statistical analysis

SPSS 25 (IBM) was utilized for data storage/handling. Continuous and ordinal variables were expressed as median and inter-quartile range. Kruskall-Wallis test used to compare continuous and ordinal data. Categorical data, summarized by count/proportions and Pearson-χ^2^ used for comparison. Collagen deposition data was compared (Welch t-test(differing variances) and confirmed normality(Shapiro-Wilk test)). Preprocessing eliminated metabolites with insufficient variation (relative standard deviation <15%) or detection frequency (<20% of mice/group) and imputed missing data with the minimum observed value per-compound^46^. Curated metabolite data was subjected to RF (randomForest Package R3.4.3, R-Project) to identify a refined profile of the top 5% of metabolites important to class separation as measured by mean decrease accuracy. Random Forests was re-run using this refined profile to assess its classification ability as previously described^17,47^. PCA used for feature projection and data visualization to identify relationships between correlated metabolites.

## Supplementary information

Supplemental table.

## Data Availability

Additional data available upon reasonable request to the corresponding author.
