# Author Correction: World Trade Center-Cardiorespiratory and Vascular Dysfunction: Assessing the Phenotype and Metabolome of a Murine Particulate Matter Exposure Model

**DOI:** 10.1038/s41598-021-95856-0

**Published:** 2021-08-05

**Authors:** Arul Veerappan, Assad Oskuei, George Crowley, Mena Mikhail, Dean Ostrofsky, Zakia Gironda, Sandhya Vaidyanathan, Youssef Zaim Wadghiri, Mengling Liu, Sophia Kwon, Anna Nolan

**Affiliations:** 1 Department of Medicine, Division of Pulmonary, Critical Care and Sleep Medicine, NYU, School of Medicine, NY, New York, NY USA; 2 Bureau of Health Services, Fire Department of New York, Brooklyn, NY USA; 3 Center for Advanced Imaging Innovation and Research (CAI2R) & Bernard and Irene Schwartz Center for Biomedical Imaging, Department of Radiology, NYU School of Medicine, NY, New York, NY USA; 4 Department of Environmental Medicine, New York University, School of Medicine, NY, New York, NY USA; 5 Department of Population Health, Division of Biostatistics, NYU School of Medicine, NY, New York, NY USA

Correction to: Scientific Reports, 10.1038/s41598-020-58717-w, published online 21 February 2020

The original version of this Article contained an error in the Acknowledgments section.

“This work was supported, in part, by NHLBI R01HL119326 and CDC/NIOSH U01-OH011300 and was also performed at the Preclinical Imaging Laboratory, a shared resource partially supported by the Laura and Isaac Perlmutter Cancer Center Support Grant NIH/NCI 5P30CA016087 and NIBIB Biomedical Technology Resource Center Grant NIH P41 EB017183.”

now reads:

“This work was supported, in part, by NHLBI R01HL119326, CDC/NIOSH U01-OH011300, the Stony Wold-Herbert Fund and was also performed at the Preclinical Imaging Laboratory, a shared resource partially supported by the Laura and Isaac Perlmutter Cancer Center Support Grant NIH/NCI 5P30CA016087 and NIBIB Biomedical Technology Resource Center Grant NIH P41 EB017183.”

The original Article has been corrected.

