# Supplementary material for: World Trade Center-Cardiorespiratory and Vascular Dysfunction: Assessing the Phenotype and Metabolome of a Murine Particulate Matter Exposure Model
Source: Sci Rep. 2020 Feb 21;10:3130. doi: 10.1038/s41598-020-58717-w (PMC7035300; doi:10.1038/s41598-020-58717-w)
Supplement: Supplementary file 1 — Supplemental table. [file 41598_2020_58717_MOESM1_ESM.pdf]

World Trade Center-Cardiorespiratory and Vascular Dysfunction: Assessing the Phenotype and Metabolome of a Murine Particulate Matter Exposure Model

Arul Veerappan[1]; Assad Oskuei[1]; George Crowley[1,2]; Mena Mikhail[1]; Dean Ostrofsky[1]; Zakia Gironda[3]; Sandhya Vaidyanathan[1]; Youssef Zaim Wadghiri[3]; Mengling Liu[4,5]; Sophia Kwon[1]; Anna Nolan[1,2,4].

[1] Department of Medicine, Division of Pulmonary, Critical Care and Sleep Medicine, NYU, School of Medicine, NY, NY

[2] Bureau of Health Services, Fire Department of New York, Brooklyn, NY

[3] Center for Advanced Imaging Innovation and Research (CAI2R) & Bernard and Irene Schwartz Center for Biomedical Imaging, Department of Radiology, NYU School of Medicine, NY, NY

[4] Department of Environmental Medicine, New York University, School of Medicine, NY, NY

[5] Department of Population Health, Division of Biostatistics, NYU School of Medicine, NY, NY

[o] ORCiD https://orcid.org/0000-0002-0631-1171

Table 1. Echocardiography: 24-Hours and 1-Month After WTC-PM Exposure in Comparison to Their Controls

	Parameters	24-Hours		1-Month	
		WTC-PM	PBS	WTC-PM	PBS
PA	**PAT** (ms)	**10.83** (3.15)**	**17.57** (3.25)	**11.64** (3.01) **	**17.34** (3.02)
	**PET** (ms)	**56.37** (5.82)	**51.11** (6.91)	**56.55** (10.95)	**56.21** (6.06)
	**PAT/PET**	**0.19** (0.06)***	**0.34** (0.03)	**0.21** (0.03)***	**0.29** (0.02)
	**PA VTI** (mm)	**14.04** (3.11)	**16.12** (2.88)	**17.49** (2.71)	**19.45** (4.45)
	**Peak Pressure** (mmHg)	**0.87** (0.28)*	**1.22** (0.33)	**1.06** (0.22)*	**1.32** (0.20)
	**MAP** (Common) (mmHg)	**74.22** (1.41)**	**70.81** (1.65)	**73.76** (1.35) **	**71.46** (0.95)
	**MAP** (PAT<120ms) (mmHg)	**83.41** (1.95)**	**79.02** (2.02)	**82.79** (1.87) **	**79.62** (1.31)
	**Peak Velocity** (mm/s)	**471.10** (65.98)	**537.75** (71.5)	**511.50** (57.53)*	**573.60** (44.9)
	**Mean Velocity** (mm/s)	**267.13** (39.95)*	**314.70** (39.06)	**312.30** (28.73)	**336.50** (30.4)
	**Peak Gradient** (mmHg)	**0.86** (0.24)*	**1.15** (0.29)	**1.08** (0.18)	**1.29** (0.24)
	**Mean Gradient** (mmHg)	**0.29** (0.08)*	**0.40** (0.09)	**0.39** (0.06)	**0.46** (0.08)
LV	**HR** (beats/min)	**474.00** (23.13)	**478.37** (38.8)	**450.78** (31.24)	**476.05** (13.9)
	**CO** (ml/min)	**10.74** (2.75)*	**13.99** (4.26)	**13.78** (1.72)**	**17.16** (1.48)
	**SV** (μl)	**22.58** (5.45)*	**29.01** (8.01)	**30.67** (4.02)	**31.57** (8.63)
	**EF** (%)	**74.06** (11.59)	**79.71** (9.63)	**66.01** (6.18)	**71.26** (9.47)
	**FS** (%)	**42.80** (8.75)	**48.06** (9.1)	**35.70** (4.69)	**40.47** (9.41)
	**Volume**$_{Systolic}$ (μl)	**7.50** (2.83)	**8.16** (7.2)	**16.30** (4.93)	**13.80** (5.93)
	**Volume**$_{Diastolic}$ (μl)	**30.08** (6.23)	**37.17** (13.53)	**46.97** (7.78)	**45.37** (14.0)
	**Diameter**$_{Systolic}$ (mm)	**1.59** (0.24)	**1.58** (0.45)	**2.18** (0.27)	**1.99** (0.49)
	**Diameter**$_{Diastolic}$ (mm)	**2.80** (0.24)	**3.03** (0.42)	**3.37** (0.24)	**3.29** (0.48)
Aorta	**Aortic Root Diameter** (mm)	**1.41** (0.05)	**1.42** (0.08)	**1.60** (0.04)	**1.58** (0.08)
	**Diameter**$_{Systolic}$ (mm)	**1.34** (0.08)	**1.33** (0.11)	**1.15** (0.18)	**1.11** (0.23)
	**Diameter**$_{Diastolic}$ (mm)	**1.63** (0.00)	**1.67** (0.04)	**1.61** (0.12)	**1.42** (0.15)
	**AoV AAT** (ms)	**13.47** (2.81)	**19.03** (5.81)	**13.29** (1.59)****	**17.75** (4.37)
	**AoV AET (ms)**	**55.69** (3.47)*	**50.69** (1.61)	**59.35** (6.11)*	**50.97** (3.82)
	**AoV AAT/AET** (ratio)	**0.24** (0.04)*	**0.37** (0.11)	**0.22** (0.01)****	**0.35** (0.09)
	**AoV VTI** (mm)	**22.27** (4.88)*	**15.82** (4.63)	**24.30** (4.31)*	**18.04** (3.68)
	**AoV Peak Velocity** (mm/s)	**679.70** (119.6)	**528.93** (127.6)	**688.30** (73.01)*	**824.54** (109.92)
TV	**A Peak** (mm/s)	**225.41** (2.35)	**216.43** (16.05)	**323.80** (34.48)**	**248.45** (21.49)
	**E Peak** (mm/s)	**150.85** (3.95)	**152.75** (3.35)	**221.03** (9.08)****	**151.25** (10.32)
	**E/A** (ratio)	**0.67** (0.01)	**0.71** (0.04)	**0.72** (0.03)*	**0.61** (0.02)
RV	**Internal Diameter** (mm)	**1.02** (0.09)	**0.97** (0.15)	**0.84** (0.04)**	**0.98** (0.04)

Mean ± SD, n=8/group,

*P<0.05 **P<0.01 ***P<0.001 ****P<0.0001 and others are not significant.

Abbreviations: PA Pulmonary Artery; **LV** Left Ventricle; **RV** Right Ventricle; **PAT** Pulmonary Acceleration Time; **PET** Pulmonary Ejection Time; **VTI** Velocity Time Integral; **HR** Heart Rate; **CO** Cardiac Output; **EF** Ejection Fraction; **FS** Fractional Shortening; **AoV** Aortic Valve; **TV** Tricuspid Valve; **E Peak** The Peak Velocity of E (early) Wave; **A Peak** The Peak Velocity of A (atrial/after) Wave

Supplemental Table Legend

Table 1. Echocardiographic results, 24-hrs and 1-M after WTC-PM and PBS Exposure. All values Mean ± SD, n=8, $^{*}p<0.05$; $^{**}p<0.01$; $^{***}p<0.001$; $^{****}p<0.0001$ and others are not significant.
